# Functional Analysis of *Hyaloperonospora arabidopsidis* RXLR Effectors

**DOI:** 10.1371/journal.pone.0110624

**Published:** 2014-11-06

**Authors:** Michiel J. C. Pel, Paul C. A. Wintermans, Adriana Cabral, Bjorn J. M. Robroek, Michael F. Seidl, Jaqueline Bautor, Jane E. Parker, Guido Van den Ackerveken, Corné M. J. Pieterse

**Affiliations:** 1 Plant-Microbe Interactions, Department of Biology, Faculty of Science, Utrecht University, Utrecht, The Netherlands; 2 Centre for BioSystems Genomics, Wageningen, The Netherlands; 3 Ecology and Biodiversity, Department of Biology, Faculty of Science, Utrecht University, Utrecht, The Netherlands; 4 Theoretical Biology and Bioinformatics, Department of Biology, Faculty of Science, Utrecht University, Utrecht, The Netherlands; 5 Department of Plant-Microbe Interactions, Max Planck Institute for Plant Breeding Research, Cologne, Germany; Virginia Tech, United States of America

## Abstract

The biotrophic plant pathogen *Hyaloperonospora arabidopsidis* produces a set of putative effector proteins that contain the conserved RXLR motif. For most of these RXLR proteins the role during infection is unknown. Thirteen RXLR proteins from *H. arabidopsidis* strain Waco9 were analyzed for sequence similarities and tested for a role in virulence. The thirteen RXLR proteins displayed conserved N-termini and this N-terminal conservation was also found in the 134 predicted RXLR genes from the genome of *H. arabidopsidis* strain Emoy2. To investigate the effects of single RXLR effector proteins on plant defense responses, thirteen *H. arabidopsidis* Waco9 RXLR genes were expressed in *Arabidopsis thaliana*. Subsequently, these plants were screened for altered susceptibility to the oomycetes *H. arabidopsidis* and *Phytophthora capsici*, and the bacterial pathogen *Pseudomonas syringae*. Additionally, the effect of the RXLR proteins on flg22-triggered basal immune responses was assessed. Multifactorial analysis of results collated from all experiments revealed that, except for RXLR20, all RXLR effector proteins tested affected plant immunity. For RXLR9 this was confirmed using a *P. syringae* Δ*CEL*-mediated effector delivery system. Together, the results show that many *H. arabidopsidis* RXLR effectors have small effects on the plant immune response, suggesting that suppression of host immunity by this biotrophic pathogen is likely to be caused by the combined actions of effectors.

## Introduction

Plants possess an effective innate immune system that is activated upon recognition of microbial pathogens. Plants recognize microorganisms by detecting microbe-associated molecular patterns (MAMPs), such as bacterial flagellin, which leads to MAMP-triggered immunity (MTI) [1]. Successful pathogens evolved ingenious mechanisms to evade or suppress host immunity [2]–[4]. An important class of immune suppressive proteins produced by pathogenic microbes are the so-called effector proteins, which are often able to suppress MTI responses in susceptible hosts. Effectors are pathogen-derived proteins that facilitate infection by altering host cellular processes [5]. Plant pathogenic bacteria, fungi and oomycetes all use effector proteins during infection [6]. These effector proteins enable pathogens to colonize their host and cause disease. Numerous effector proteins are recognized by disease resistance (R) proteins, leading to effector triggered immunity (ETI). The outcome of a specific plant-pathogen interaction depends on the specific repertoires of *R* genes and effectors in the plant and pathogen.

Oomycetes are fungus-like eukaryotic microorganisms related to brown algae [7]. Oomycete plant pathogens cause some of the most destructive plant diseases in the world. The potato late blight pathogen *Phytophthora infestans*, for example, is responsible for a loss in crop yields of over €1 billion a year in the European Union alone. Other oomycetes, such as *Phytophthora sojae* and *Peronosclerospora sorghi,* also cause severe damage to the economically important crops soybean and sorghum, respectively [8]–[10]. Research on oomycete pathogens gained new impetus in the early-90s with genomic research and the cloning of oomycete avirulence (*Avr*; effectors defined initially as recognized by specific host *R* genes) and pathogenicity genes [11]–[14]. The discovery of conserved motifs in oomycete Avr proteins, coupled with whole genome sequencing of several oomycete pathogens resulted in the identification of hundreds of putative Avr/effector proteins [15]–[18]. Oomycete effector proteins can broadly be divided into two groups: effectors that are released into the plant apoplast and effectors that are delivered into the host cytoplasm. Host-translocated effectors were initially identified because of their avirulence function, which causes them to trigger a hypersensitive response in plants that carry the matching *R* gene. With the aid of evolutionary genomics approaches, two main types of oomycete host-translocated effectors are currently recognized, Crinklers and RXLR-effectors [19]–[23].

In the last decade, a large number of RXLR effector proteins have been identified [24]. ATR1 and ATR13 are two effectors that are produced by the downy mildew pathogen *Hyaloperonospora arabidopsidis*, a pathogenic oomycete that infects *Arabidopsis thaliana* [19], [22]. ATR1 and ATR13 each have an N-terminal signal peptide followed by the highly conserved RXLR amino acid sequence motif. This motif codes for an arginine (R), a random amino acid (X), a leucine (L) and another arginine and can be found in effector proteins of different oomycetes, suggesting that it is important for their function. The RXLR motif is sometimes followed by the less conserved dEER motif which consists of two glutamic acid residues and an arginine residue, preceded by an optional aspartic acid residue [22]. Apart from its presence in oomycetes, an RXLR-like motif has been found in proteins identified in malaria parasites (*Plasmodium* species), which are also translocated into host cells. In *Plasmodium* species this motif is called the HT/PEXEL (host-targeting/*Plasmodium* export element) motif and was demonstrated to be essential for translocating the HT/PEXEL proteins into host blood cells [25], [26]. This, together with the finding that matching R proteins often reside in the host cytoplasm, led to the hypothesis that the RXLR motif has a role in protein delivery into host cells. This was confirmed by the finding that the RXLR-EER motif of the *P. infestans* effector protein Avr3a could target *Plasmodium* proteins into the erythrocyte cytoplasm [27]. Furthermore, substitutions of the RXLR and dEER motifs of effectors Avr1b and Avr3a with other residues blocked translocation of these proteins into plant cells [28], [29], indicating that the RXLR motif is essential for cytoplasmic delivery of these effectors. Kale *et al*. [30] proposed that the RXLR domain binds to the phospholipid phosphatidylinositol-3-phosphate (PIP), which is then followed by endocytosis. However, Yaeno *et al*. [31] showed that a positively charged patch in the effector domain of AVR3a, and not the RXLR domain, is involved in PIP binding [23], [24], [30], [31]. Hence, the exact role of the RXLR motif in protein translocation remains unclear [32].

Another important aspect of oomycete effector biology is the identification of effector host targets. With over 130 putative *RXLR* effector genes in the *H. arabidopsidis* genome, over 350 predicted RXLR effectors in *P. ramorum* and *P. sojae,* and more than 550 *RXLR* effector sequences in the genome of *P. infestans,* assigning functions to all of them is an enormous challenge [16]–[18], [23]. A number of screens to determine virulence and/or avirulence functions of predicted RXLR effectors have already been undertaken. Screening of large numbers of putative RXLR effector proteins for their ability to trigger specific cell death responses resulted in the identification of only a small number of RXLR proteins with an avirulence function [33]–[37]. In contrast, the search for RXLR effectors that contribute to virulence resulted in more positive candidates. In a screen of 169 putative effectors of *P. sojae,* most were able to suppress programmed cell death responses [33]. In another study a set of 64 RXLR effector candidates of *H. arabidopsidis* isolate Emoy2 were tested for their ability to suppress callose deposition and growth of the bacterial pathogen *Pseudomonas syringae* in 12 *A. thaliana* accessions. A total of 43 RXLR proteins were found to enhance bacterial growth and 35 suppressed callose deposition [36], confirming the notion that RXLR effector proteins function by modulating host immunity. An example of this is RxL44 of *H. Arabidopsis* Emoy2 that is able to downregulate salicylic acid-triggered defense responses by targeting the host’s Mediator subunit 19A for degradation [38].

Cabral *et al.* [39] recently described a set of 18 RXLR-containing proteins that were produced by *H. arabidopsidis* isolate Waco9 during infection of *A. thaliana*. These putative effectors were identified from Expressed Sequence Tags (ESTs), which were obtained from leaves of the highly susceptible *A. thaliana* Ws *eds1-1* mutant infected with the *H. arabidopsidis* strain Waco9. Additional sequencing of alleles of the 18 *RXLR* genes in other *H. arabidopsidis* isolates revealed signs of diversifying selection, supporting a putative effector role of the identified RXLR proteins. Furthermore, Cabral *et al.* [39] showed that one of the identified RXLRs, RXLR29, is able to suppress MTI and enhances disease susceptibility to *P. syringae* in *A. thaliana*. In order to identify potential functions of the other RXLRs we undertook intensive screening of 13 of the 18 *RXLR* genes described by Cabral *et al.* (2011), and assessed their effect on host immunity. Transgenic *A. thaliana* plants expressing *H. arabidopsidis RXLR* genes were generated and screened for enhanced susceptibility to several different pathogens. In addition, the *RXLR* expressing lines were checked for altered MTI responses. Furthermore, we used the EDV-system, which exploits the bacterial type III secretion system to secrete proteins into host cells, to deliver the RXLR proteins to *A. thaliana* leaf cells in order to confirm actions of selected RXLRs in suppressing MTI.

## Materials and Methods

### Sequence analysis

To assess sequence conservation in the 18 RXLR effector proteins identified by Cabral *et al*. [39], protein sequences were aligned using CLC Main Workbench software (www.clcbio.com), resulting in the identification of 4 distinct groups. Subsequently, the members within each group were aligned. A similarity-score per position of the multiple sequence alignment was derived based on the similarity of each amino acid and the five amino acids upstream and downstream to the consensus sequence of that group. The conservation-score per site was converted into a graphical representation via Matrix2png [40].

To identify (for Emoy2 RXLR proteins) and confirm (for Waco9 RXLR proteins) clusters of similar N-terminal RXLR effector regions, 130 Emoy2 RXLR proteins were retrieved alongside 18 Waco9 RXLR sequences from Baxter *et al*. [18] and Cabral *et al*. [39], respectively, from which the N-terminal 60 amino acids were extracted. The similarity between these sequences was established using BLASTp [41] with an e-value cutoff of 1e-5. Clusters of similar N-terminal regions were formed based on the similarity using the MCL clustering algorithm [42], [43] with an inflation value of 2.

### Cultivation of plants

*A. thaliana* accession Col-0 and Col-0 mutants *npr1-1* [44], *fls2* (SALK_141277) [45] and *eds1-2* [46] were used. Seeds of *A. thaliana* lines were sown on quartz sand. Two-week-old seedlings were transferred to 60-mL pots containing a sand-potting soil mixture that had been autoclaved twice for 20 min with a 24-h interval. *A. thaliana* used for *H. arabidopsidis* and *Phytophthora capsici* inoculations were sown directly on this sand-potting soil mixture. Plants were cultivated in a growth chamber with a 10-h day (200 µE·m^−2^·s^−1^ at 21°C) and a 14-h night (20°C) cycle at 70% relative air humidity. Plants were supplied with modified half-strength Hoagland nutrient solution once a week [47], as described [48].

### Construction of transgenic *A. thaliana* lines

The open reading frames of *H. arabidopsidis* isolate Waco9 *RXLR* genes without the predicted signal peptide [39] were cloned in pENTR/D-TOPO (Invitrogen) and transferred by LR recombination (Invitrogen) into the Gateway destination binary vectors pAMPAT-GW with *Cauliflower mosaic virus 35S* promoter driven expression. Constructs were transferred to *Agrobacterium tumefaciens* strain GV3101 (pMP90RK) and transformed into *A. thaliana* Col-0 using the floral-dip method [49]. Transformants were selected by spraying T1 progeny with BASTA Finale SL14 (Bayer CropScience BV, Mijndrecht, The Netherlands) according to the manufacturers instruction. From 1∶3 segregating T2 lines, homozygous T3 lines were obtained for further testing.

The *in planta* expression of the Waco9 *RXLR* genes was verified using semi-quantitative RT-PCR. Three-week-old plants were harvested and total RNA was isolated as described by Van Wees et al. [50]. Fermentas RevertAid H minus Reverse Transcriptase (Fermentas, St. Leon-Rot, Germany) was used to synthesize cDNA. The cDNA was amplified for 20, 25 or 30 cycles using gene-specific primer pairs (Table S1).

### Cultivation of pathogens and pathogen inoculation

*H. arabidopsidis* isolate Waco9 was maintained on susceptible Col-0 plants as described [51]. Sporangia were obtained by washing seedlings that were densely covered by sporangiophores in water. The obtained suspension was filtered using Miracloth and diluted with water to a concentration of 50 spores·µL^−1^. *A. thaliana* seedlings of 14 days old were sprayed with the spore suspension and dried for 2 h. Subsequently, the plants were placed at 16°C, 9-h day (100 µE·m^−2^·s^−1^), 15-h night and 100% relative humidity for 6 days. Disease was scored by determining the number of sporangiophores per plant [52].

*P. capsici* LT3112 [53] was grown on V8 agar plates for one week at 21°C, 10-h light and 14-h dark. To collect zoospores, overgrown agar slices were placed in 10 mL sterile dH_2_O for 1 h at 20°C. Subsequently, the dH_2_O was replaced with fresh dH_2_O and the agar slices were kept at 24°C overnight followed by 1 hour at 4°C. The dH_2_O was checked for zoospores and diluted to a concentration of 50 zoospores·µL^−1^. Plants were sprayed with the zoospore suspension and kept at 21°C, 100% air humidity and complete darkness for 24 h after which the plants were transferred to standard growing conditions with 100% air humidity. Six days after inoculation disease severity was determined by scoring the percentage of diseased leaves per plant. Leaves were scored as diseased when macroscopically visible necrotic lesions were present [53].

*P. syringae* pv. *tomato* DC3000 strains were cultured overnight in King’s medium B at 28°C. For growth of *P. syringae* pv. *tomato* DC3000 ΔCEL [54], 50 µg·mL^−1^ rifampicin and 100 µg·mL^−1^ spectinomycin was added to the medium. Bacterial cells were collected by centrifugation (4000 rpm, 10 min) and for dipping experiments the bacteria were resuspended and diluted to a final concentration of 2.5·10^−7^ cfu·mL^−1^ in 10 mM MgSO_4_ containing 0,015% (v/v) Silwet L-77. Leaves of 5-week-old *A. thaliana* plants were dipped in this bacterial suspension, after which the plants were placed at 100% relative air humidity. Disease levels were assessed at 3 or 4 d post inoculation by determining the percentage of leaves showing symptoms. Leaves were scored as diseased when necrotic or water-soaked lesions surrounded by chlorosis were visible. Disease index was determined as described [48].

### Flg22-mediated growth reduction assay

Sterile *A. thaliana* seeds were placed in 24-well plates containing 1 mL of MS medium (4,4 g·L^−1^ MS, 10 g·L^−1^ sucrose, pH 5.7). Ten seeds were placed in each well and 3 wells were used per line per treatment. Depending on the treatment, flg22 was added to a final concentration of 50 nM or 500 nM. After 2 days at 4°C the plates were placed at standard plant growth conditions (as described under cultivation of plants) for 10 days. Subsequently, the fresh weight of the total plant biomass per well was measured and the number of plants in each well was determined as described [2].

### Statistical analysis

Redundancy analyses (RDA) were applied to the phenotypic data obtained in the different experiments of this study (Hellinger transformed within treatment) to perform a multifactorial test on the effects of the RXLR overexpressors on different components of the plant immune system (coded as binary variables). The significance of the models and of each explanatory variable included in the models was tested using 1000 permutations. Phenotype scores from the significant (Pr(>F) <0.05) RDA axes (RDA1 and RDA2) were used in RDA ordination to perform hierarchical cluster analysis according to the ‘Ward method’, and the resulting dendrogram was projected in the RDA ordination space. This allows identification of the main discontinuities among groups and/or genotypes described by all descriptors [55]. Identification of groups was done using the pvrect function in the *pvclust* package in the software package R. All multivariate analyses were performed with the software package R 2.15.2 “Trick or Treat” [56] using the *vegan* [57] and *pvclust* [58] packages. For the heatmap, the relative averages of the different experiments were placed in a matrix and this matrix was converted into a heatmap using Matrix2png [40].

### Callose staining and microscopic analysis

Analysis for callose deposition was performed mainly as described [39], [59]. In brief, leaves of 5-week-old *A. thaliana* accession Col-0 plants were pressure-infiltrated with a 2-ml syringe containing a bacterial suspension consisting of 1×10^8^ cfu·mL^−1^ *P. syringae* pv. *tomato* DC3000 ΔCEL in 10 mM MgSO_4_. A total of 80 leaf samples were taken for callose staining 12 to 14 h after infiltration. Leaves were cleared with 100% ethanol, re-hydrated and stained with aniline blue (0.05% in phosphate buffer pH 8.0) for 24 h. Samples were analyzed with an Olympus AX70 microscope using an UV filter. Callose spots were counted using the ImageJ software (http://rsb.info.nih.gov/ij/) [60].

## Results

### RXLR effectors can be separated into groups based on their conserved N-termini

**Figure 1:**
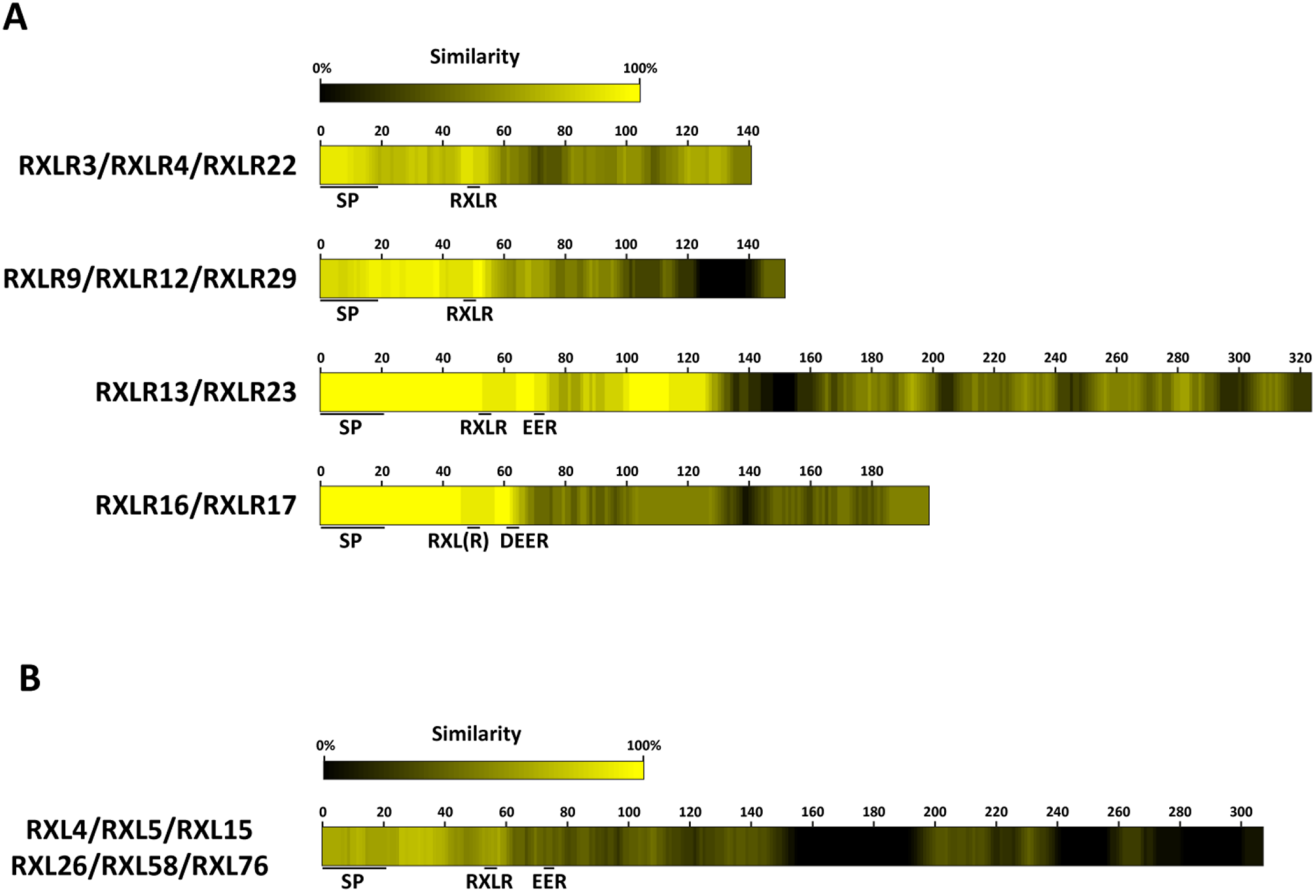
N-terminal sequence conservation of *H. arabidopsidis* RXLR proteins. (A) The amino acid sequences of the 18 RLXR proteins of *H. arabidopsidis* isolate Waco9 identified by Cabral *et al*. [38] were grouped using BLASTp on the N-terminal 60 amino acids of these proteins. Color schemes show for each amino acid the similarity within the 11 amino acid region surrounding the specific amino acid. (B) N-terminal 60-amino acid regions of 130 Emoy2 RXLR proteins [18] were checked for similarity using BLASTp, leading to the identification of 23 groups containing at least two Emoy2 RXLRs. An alignment of the members of one of these identified groups is shown as example. RXL4, RXL5, RXL15, RXL26, RXL58, RXL76 are aligned and this group includes the homologs of RXLR13 (RXL76) and RXLR23 (RXL4) of *H. arabidopsidis* Waco9. Alignments are depicted as in (A).

RXLR proteins consist of an N-terminal signal peptide followed by an RXLR domain and a C-terminal effector domain. Based on their amino acid sequences, the 18 RXLR proteins identified by Cabral *et al.* [39] could be divided in four groups of two or three RXLR proteins each, and eight RXLR proteins that showed no similarity to the other RXLRs. Strikingly, within each of the four groups the N-terminus is highly conserved while the C-terminus is very divergent (Figure 1A). To investigate whether amino acid sequence conservation in the N-terminus of RXLR proteins is a common phenomenon, we aligned all 134 identified RXLR sequences in the genome of the sequenced *H. arabidopsidis* isolate Emoy2 [18]. Based on the first 60 amino acids of each RXLR protein, around 60 percent of the RXLRs can be placed in a group with at least one other RXLR protein and in most cases the similarity between proteins within one group is limited to the N-terminus (data not shown). In Figure 1B the amino acid sequence conservation pattern of the six *H. arabidopsis* RXLRs of isolate Emoy2 [18] that group with RXLR13 and RXLR23 from Waco9 is shown. The N-termini show an amino acid sequence similarity of 60%–80%, while the sequence similarity in the C-terminal parts is relatively low. Thus, conservation of the N-terminal seems to be common for RXLR proteins in *H. arabidopsidis*.

### *In planta* expression of Waco9 RXLR effectors

**Figure 2:**
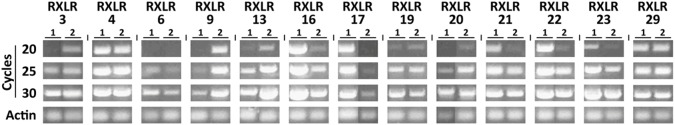
Expression levels of *H. arabidopsidis RXLR* genes in *A. thaliana.* Semi-quantitative analysis of the expression levels of the *RXLR* transgenes from *H. arabidopsidis* Waco9 in *A. thaliana* accession Col-0. Expression levels were assessed in two independent transgenic lines using *RXLR* gene-specific primers. Depicted are ethidium bromide-stained agarose gels with PCR products after 20, 25 or 30 cycles of PCR amplification. The PCR product of the *A. thaliana actin* gene (At3g18780) was used as internal control (25 cycles of PCR amplification).

The *RXLR* gene transcripts of *H. arabidopsidis* identified by Cabral *et al.* [39] are expressed during infection, suggesting a role in pathogen virulence. While for other oomycete pathogens transformation protocols have been established [12], [61], [62], it is currently still not possible to transform *H. arabidopsidis*. Hence, in order to investigate the function of *H. arabidopsis* effector proteins in the infection process, *A. thaliana* plants were transformed to constitutively express a single *RXLR* effector gene. Of the 18 *RXLR* effector genes identified by Cabral *et al.* [39], the coding region without the signal peptide of 13 was successfully cloned behind the constitutive *35S CaMV* promoter and transformed into *A. thaliana* (Table 1). Independent lines of *RXLR* transgenes that were segregating for a single transgene and had different levels of expression were selected (Figure 2). To check if expression of the *RXLR* effector genes affected plant growth and development, the rosette diameters and leaf morphology were monitored during a growth period of five weeks. In none of the transgenic lines did expression of the *RXLR* gene lead to a noticeably altered plant phenotype (data not shown).

**Table 1:** Sequence features of successfully cloned *RXLR* genes of *H. arabidopsidis* isolate Waco9.

	RXLR	Emoy2^1^	Remark	Size (AA)	RXLR dis.^2^	EER dis.^3^	Homologs
1	RXLR3			129	29	-	*H. arabidopsidis*
2	RXLR4			134	29	-	*H. arabidopsidis*
3	RXLR6	HaRXL80		129	27	44	*H. arabidopsidis*
4	RXLR9	HaRXL78		150	28	-	*H. arabidopsidis*
5	RXLR13	HaRXL76		286	32	49	*H. arabidopsidis*
6	RXLR16	HaRXL30; HaRXL79		198	28	41	*H. arabidopsidis*
7	RXLR17	HaRXL42	RXLQ	135	28	41	*H. arabidopsidis*
8	RXLR19			299	31	-	*H. arabidopsidis*
9	RXLR20	HaRXL10		241	23	-	*H. arabidopsidis*
10	RXLR21	HaRXL37; HaRXL75		115	29	44	Oomycetes
11	RXLR22			137	29	-	Waco9 specific
12	RXLR23	HaRXL4		304	32	49	*H. arabidopsidis*
13	RXLR29			132	28	-	*H. arabidopsidis*

1 Gene ID of RXLRs from Emoy2 [18]).

2 Number of amino acid to signal peptide cleavage site.

3 When present, number of amino acid to signal peptide cleavage site.

4 Homologs present in this group, adapted from Cabral *et al.* [18].

### Expression of single Waco9 *RXLR* effector genes does not affect basal resistance against *H. arabidopsidis*

**Figure 3:**
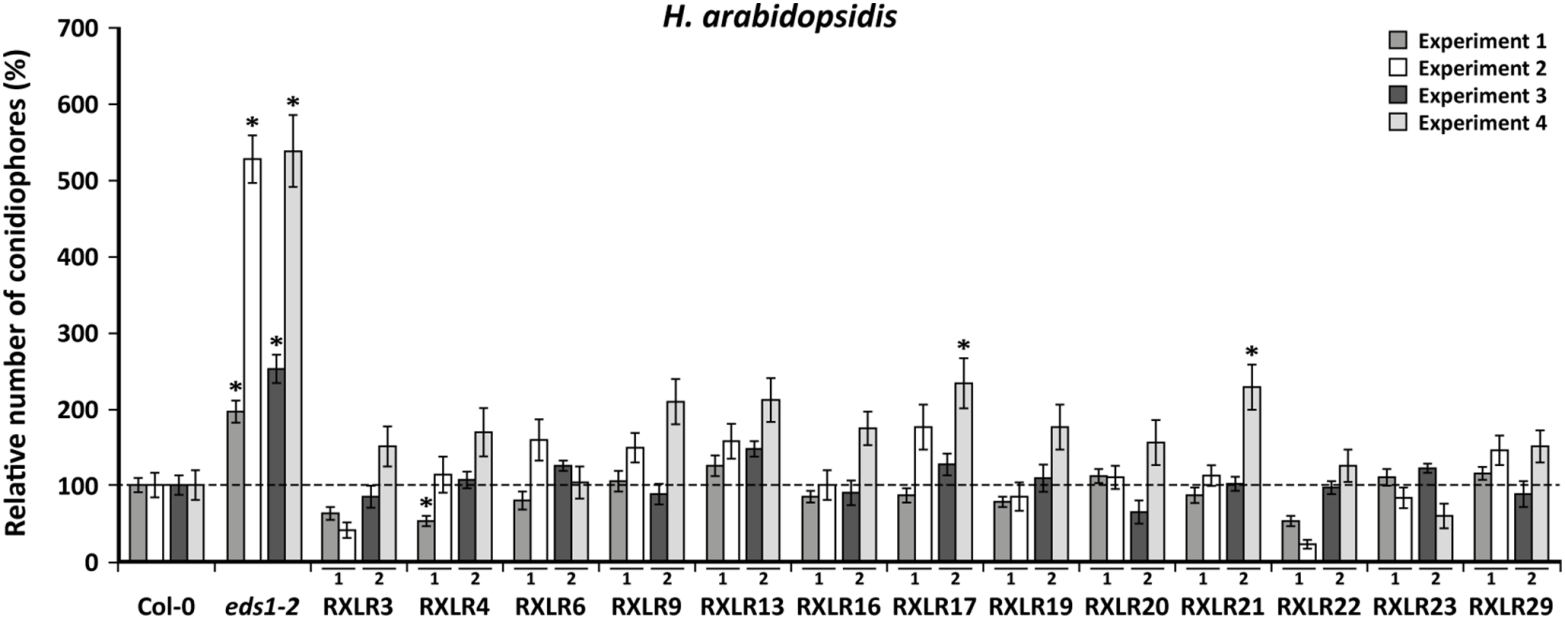
Effect of ectopic expression of Waco9 *RXLR* genes on the level of resistance against *H. arabidopsidis*. In four experiments, two independent overexpressing lines of each of the 13 Waco9 *RXLR* genes was tested twice for the level of resistance against *H. arabidopsidis* Waco9. Two-week-old plants were spray inoculated and 6 days later the number of conidiophores per plant was determined. In each experiment the number of conidiophores on Col-0 is set at 100%. Subsequently, the number of conidiophores in all other lines is given relative to Col-0 in the same experiment. The enhanced susceptible mutant *eds1-2* was included as a positive control. Results represent mean ± SEM (*n* = 18) and asterisks indicate significant differences (ANOVA and Fisher’s LSD corrected for type I errors; *p*<0.05).

To test whether overexpression of any of the single Waco9 *RXLR* genes in *A. thaliana* results in increased susceptibility to infection by the downy mildew pathogen, two-week-old *RXLR*-expressing seedlings were infected with *H. arabidopsidis* Waco9. After six days, disease levels were scored by counting the number of sporangiophores on each seedling. This was done in four independent experiments, in which two independent lines of each *RXLR* gene were tested twice. In all experiments the highly susceptible mutant Col-0 *eds1-2* [46] was included as a control. In Figure 3, the number of sporangiophores in the RXLR transgenic lines compared to wild-type Col-0 is shown for all four experiments. In all four experiments, *eds1-2* showed an enhanced susceptibility to *H. arabidopsidis* compared to Col-0. Only in experiment 1 and 4 did one or two of the 13 *RXLR* overexpressing lines permit enhanced *H. arabidopsidis* growth compared to Col-0. However, none of the *RXLR*-overexpressing lines showed a consistently altered level of disease resistance in more than one experiment. We therefore concluded that none of the 13 tested *H. arabidopsidis* RXLR effector proteins has a strong effect on the level of susceptibility to *H. arabidopsidis* when ectopically expressed *in planta*.

### Waco9 RXLRs can alter *P. capsici* resistance levels in *A. thaliana*

**Figure 4:**
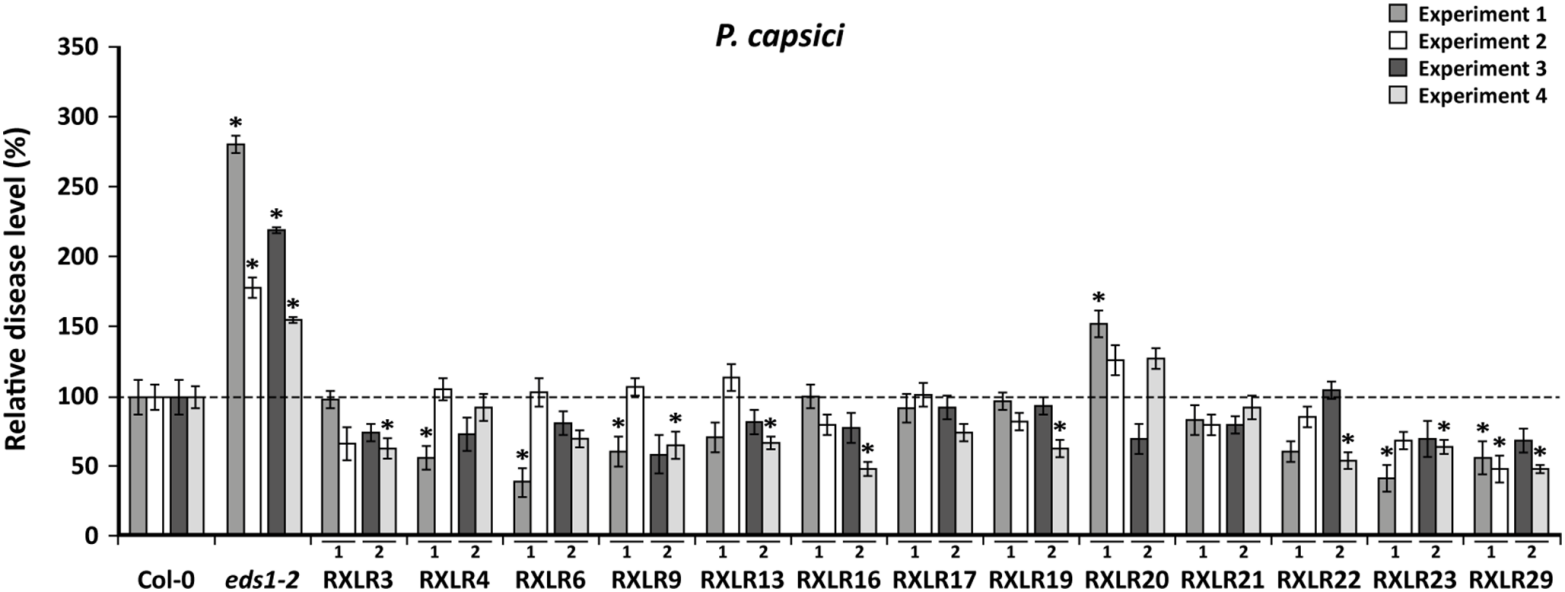
Effect of ectopic expression of Waco9 *RXLR* gene*s* on the level of resistance against *P. capsici*. In four experiments, two independent lines of each of the 13 Waco9 *RXLR* genes were tested twice for the level of resistance against *P. capsici*. Two-week-old plants were spray inoculated with *P. capsici* zoospores and the percentage of diseased leaves, showing necrotic lesions, was determined after six days. In each experiment the percentage of leaves with necrotic lesions is shown relative to that of Col-0. The enhanced susceptible mutant *eds1–2* was included as a positive control. Results represent mean ± SEM (*n* = 18) and asterisks indicate significant differences (ANOVA and Fisher’s LSD corrected for type I errors; *p*<0.05).

The *RXLR* overexpressing lines were also tested for resistance against *P. capsici.* Initially, this oomycete has a similar biotrophic lifestyle as *H. arabidopsidis*, i.e. growing intercellularly and forming haustoria that penetrate host cell walls. In contrast to *H. arabidopsidis*, *P. capsici* switches to a necrotrophic lifestyle in later stages of infection and produces a largely different set of RXLR effector proteins [17], [18], [53]. Two-week-old seedlings were infected with *P. capsici* and 6 days later the plants were scored for disease severity. Again, disease assays were performed in four independent experiments, in which two independent lines of each *RXLR* gene were tested twice. In all experiments the highly susceptible mutant *eds1-2* was included as a control. On wild-type Col-0, disease symptoms developed from small necrotic lesions to completely dead plants at day 6 after inoculation. In all experiments, disease caused by *P. capsici* developed faster on *eds1-2,* resulting in more severe symptoms at day 6 after inoculation (Figure 4). Strikingly, eleven out of thirteen *RXLR* overexpressing lines showed an altered disease phenotype upon inoculation with *P. capsici*. RXLR9- and RXLR23-overexpressing lines exhibited a lower level of disease than Col-0 in two of the four experiments, while the RXLR29 overexpressor developed significantly fewer disease symptoms in three of the four experiments. In contrast, overexpression of RXLR20 resulted in a significant increase in disease severity in one of the four experiments. Overall, RXLR9, RXLR23 and RXLR29 were able to consistently alter plant immune responses to *P. capsici* infection, suggesting a role for these effector proteins in modulating host immunity.

### Waco9 RXLRs can suppress basal resistance against *P. syringae*

**Figure 5:**
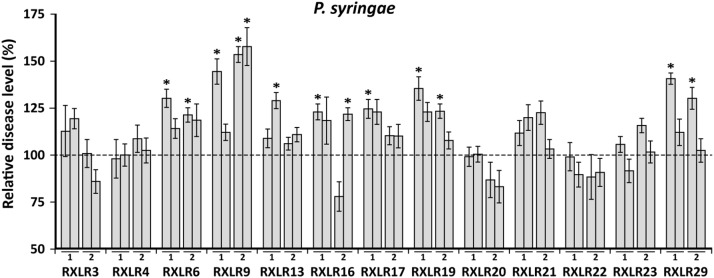
Effect of Waco9 RXLRs on the level of resistance against *P. syringae*. Five-week-old plants of Col-0, *npr1-1,* and two independent lines of each of the 13 Waco9 *RXLR-*overexpressing lines were dip-inoculated with *P. syringae* and 3 days later, the percentage of diseased leaves per plant was determined. Leaves were scored as diseased when showing necrotic or water-soaked lesions surrounded by chlorosis. In each experiment the disease level is shown relative to that of Col-0. In each experiment the enhanced susceptible mutant *npr1-1* mutant was included. Results represent mean ± SEM (*n* = 20) and asterisks indicate significant differences (ANOVA and Fisher’s LSD corrected for type I errors; *p*<0.05).

To test whether any of the RXLR proteins are able to suppress defenses that are not oomycete specific, the RXLR-overexpressing *A. thaliana* lines were inoculated with the virulent bacterial plant pathogen *P. syringae* pv. *tomato* DC3000. Disease assays were performed with two independent lines of each of the 13 Waco9 RXLR overexpressors and the level of disease severity was compared to wild-type Col-0 and the enhanced susceptible mutant *npr1-1* [44]. In Figure 5, the level of *P. syringae* disease severity of all RXLR overexpressing lines compared to Col-0 is shown. Seven of the *RXLR* overexpressors displayed altered resistance against *P. syringae* at least once, with RXLR6, RXLR16, RXLR19 and RXLR29 showing enhanced susceptibility to *P. syringae* infection in two experiments, and RXLR9 in three experiments. Overall, these results suggest that the Waco9 RXLR effectors RXLR6, RXLR9, RXLR16, RXLR19 and RXLR29 suppress immune responses to the bacterial pathogen *P. syringae.*

### Effects of Waco9 RXLRs on flg22-induced basal immune responses

**Figure 6:**
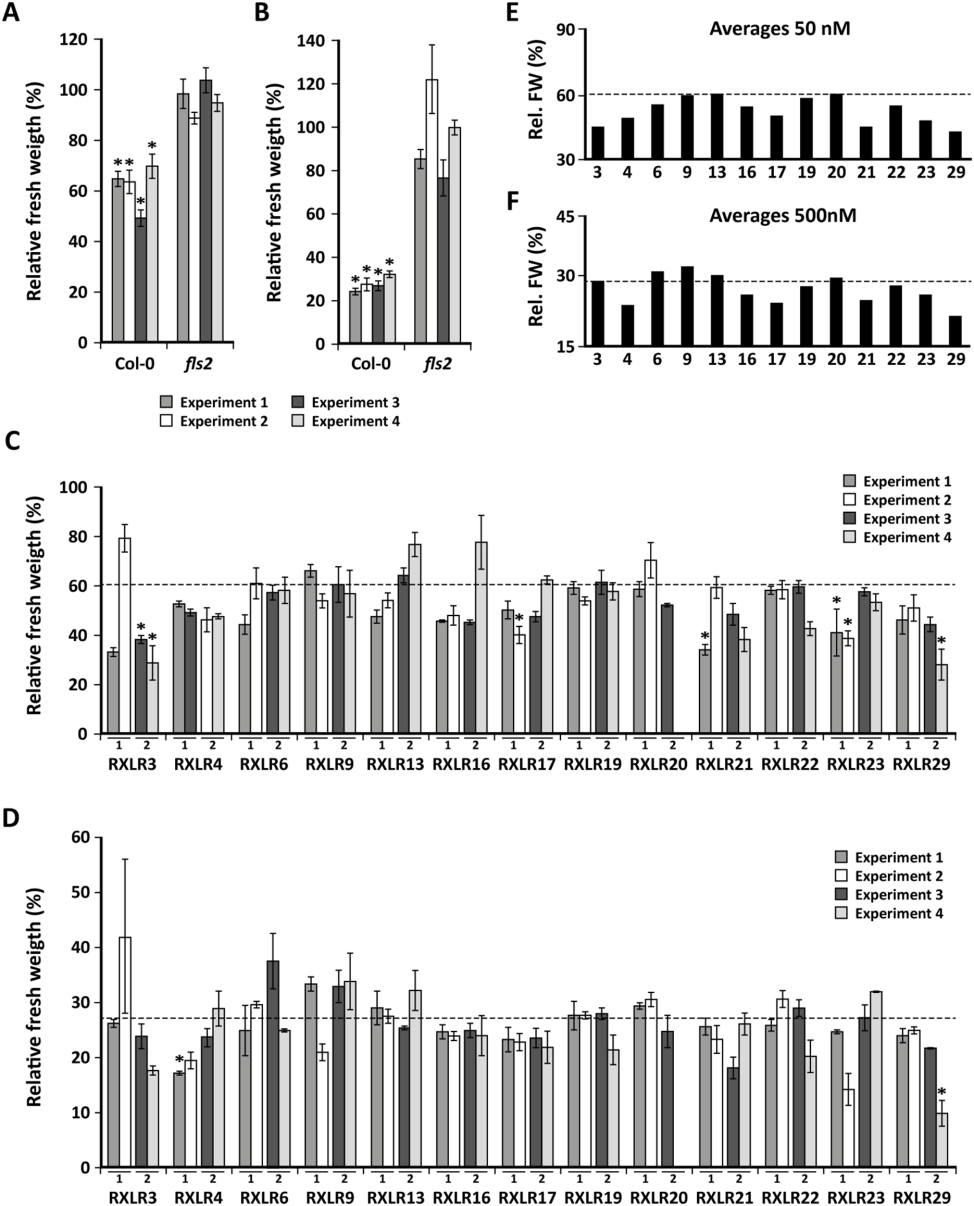
Effects of Waco9 RXLRs on flg22-induced reduction of seedling growth. In four independent experiments seedlings of Col-0, the flagellin receptor mutant *fls2*, and two independent lines of each of the 13 Waco9 RXLR overexpressors were grown on liquid MS medium in the presence or absence of 50 (A), (C) and (E) or 500 nM (B), (D) and (F) of flg22. After 10 days of growth, the fresh weight (FW) of a pool of 10 plants per line was determined. In (A) and (B) the relative FWs of Col-0 and *fls2* are depicted for the 4 independent experiments, in which the FW of the untreated plants was set at 100%. Results represent mean ± SEM (*n* = 3) and asterisks indicate significant differences between treated and non-treated plants (Students *t*-test; *p*<0.05). In (C) and (D) the relative FWs of the flg22-treated RXLR overexpressors are depicted for the 4 experiments. These relative FWs are normalized to the relative FW of untreated Col-0 (set at 100%). The dotted line shows the average relative FW of flg22-treated Col-0. Results represent mean ± SEM (*n* = 3) and asterisks indicate significant differences in relative FW compared to Col-0 (ANOVA and Fisher’s LSD corrected for type I errors; *p*<0.05). In (E) and (F) the averages of the relative FWs of the flg22-treated RXLR overexpressors are depicted (i.e. the averages of the results in (C) and (D)), again the dotted line represents the average relative FW of flg22-treated Col-0).

In the disease assays described above, we looked at the influence of the Waco9 RXLR proteins on immune responses triggered by different pathogens. In order to investigate the effect of the RXLRs specifically on MTI responses, we monitored reactions of the 13 Waco9 RXLR overexpressors to the well-characterized MAMP flg22, a 22-amino acid derivative of bacterial flagellin. Typically, treatment of *A. thaliana* seedlings with flg22 activates MTI, resulting in a growth reduction due to reallocation of resources and toxicity of defense related products [63]. The reduction in seedling growth can be used as a measure for MTI response activation [2], [64]. RXLR-overexpressing seedlings were grown in liquid MS medium containing 50 or 500 nM of flg22 and in each experiment mock-treated and flg22-treated plants of the flagellin receptor mutant *fls2* were included as controls. After 10 days of growth, the fresh weight per plant was measured and the relative change in fresh weight in flg22- versus non-treated plants was determined. Figure 6A and 6B show that treatment of Col-0 seedlings with flg22 resulted in a significant decrease in fresh weight in four independent experiments, whereas the *fls2* mutant was not responsive to flg22. In order to assess the effect of RXLR overexpression on flg22-mediated growth reduction, the average fresh weights of the RXLR overexpressors were first normalized to that of the untreated plants in the same experiment (set at 100%). Subsequently, the average fresh weights of the flg22-treated RXLR overexpressors were compared to that of flg22-treated Col-0 plants (Figures 6C and 6D). In response to treatment with 50 nM flg22, RXLR3, RXLR21, RXLR23 and RXLR29 overexpressing lines showed a significant difference in growth reduction in only one or two out of four experiments. After treatment with 500 nM flg22, RXLR4 and RXLR29 showed a significant difference in growth reduction in one out of four experiments. Surprisingly, in all statistically significant cases, the RXLR-overexpressing lines produced stronger rather than weaker flg22-induced growth inhibition compared to Col-0. Figures 6E and 6F show the averages of the four different experiments from Figures 6C and 6D, respectively. Notably, the pattern of the fresh weight averages in flg22-treated plants of each RXLR overexpressor are highly similar between plants treated with 50 nM or 500 nM flg22 and tend to be lower in the RXLR overexpressing lines than in wild-type Col-0. Taken together, these results suggest that several RXLR-overexpressing plants are slightly more sensitive to flg22-induced MTI, resulting in enhanced levels of growth reduction in flg22-treated seedlings.

### Multifactorial analysis of Waco9 RXLR-overexpressing plants

**Figure 7:**
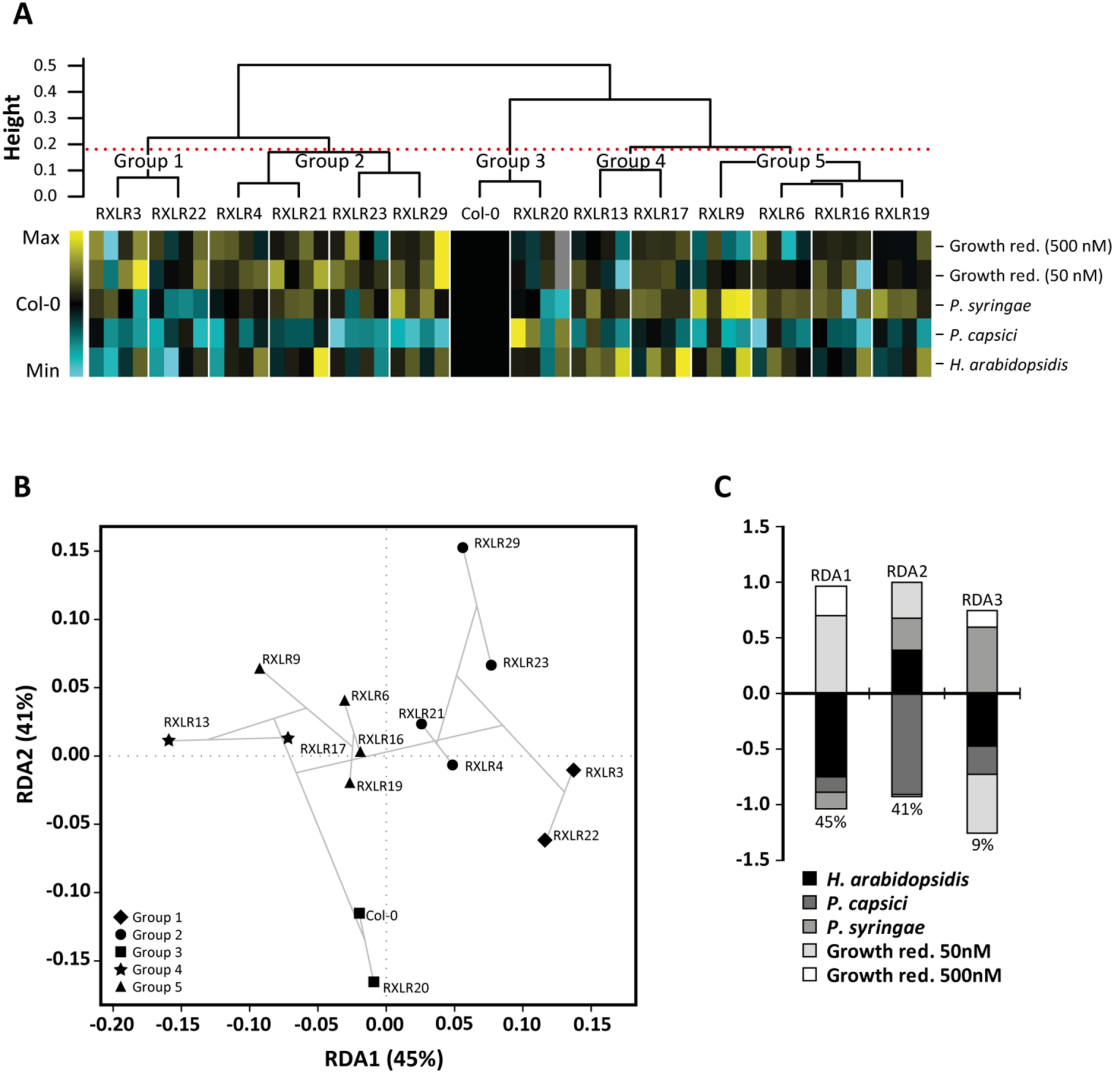
Multifactorial analysis and clustering of Waco9 RXLRs. (A) Heatmap of the relative effects of the 13 Waco9 RXLRs on flg22-mediated growth reduction and the level of resistance to *H. arabidopsidis, P. capsici,* and *P. syringae* infection. For this heatmap, the data from each experiment were converted into a score where 1 represents the maximum value, −1 represents the minimal value and the score for Col-0 was set at 0. The RXLR overexpressing lines were clustered based on the scores from the experimental results shown in Figures 3 to 6 using hierarchical clustering. The red-dotted line shows the threshold for significant difference between groups (*p*<0.05), resulting in five significantly different groups. (B) Ordination biplot generated by RDA. The statistically significant RDA axes (RDA1 and RDA2; *p*<0.05) are plotted and the five different groups based on hierarchical clustering are indicated. The dendogram based on the clustering method is projected in the ordination plot (gray lines). (C) The eigenvectors derived from the RDA. For three out of five RDA axes the amount of variation explained is shown in percentages and the contribution of each phenotype score to each RDA axes is indicated with different shades of gray. RDA4 and RDA5 are not shown since they explain only a very small part of the variation (3% and 1% respectively). The *p*-values of the RDA’s are RDA1 *p*≤0.001, RDA2 *p*≤0.001, RDA3 *p* = 0.56, RDA4 *p* = 0.95 and RDA5 *p* = 1.0.

The above-described results show that a number of Waco9 RXLR proteins, when constitutively expressed in Col-0, have relatively mild effects on different components of the plant immune system. To obtain a clearer picture of the relative importance of the effects of each of the tested RXLRs in the combined dataset, we performed a redundancy analysis (RDA) followed by a hierarchical clustering of the combined data of all the experiments performed with the RXLR overexpressors. To this end, data from all the experiments were transformed to a value between −1 and 1, with −1 being the minimum value, 1 the maximum value and 0 the score obtained for Col-0 for each treatment. Then, a heatmap of all the results from the different experiments was produced and the RXLR overexpressors were clustered using hierarchical clustering (Figure 7A). This clustering led to identification of five distinct groups of RXLR proteins (p<0.05; Figure 7A). Only *A. thaliana* constitutively expressing RXLR20 cluster together with Col-0 and thus RXLR20 does not appear to influence the tested defense responses. All other RXLRs cluster with one or three other RXLRs. The ordination biplot generated by RDA confirms the hierarchical clustering (Figure 7B). Eigenvectors derived from the RDA (Figure 7C) indicate that 45% of the variation is explained by RDA1 with growth reduction after treatment with 50 nM flg22 as a positive contributor, and *H. arabidopsidis* infection as the major negative contributor. RDA2 explains another 41% of the phenotypic variation, with *P. capsici* infection as the main contributor. Taken together, the combined results of the five different treatments show that all tested Waco9 RXLRs, except for RXLR20, have an effect on one or more components of the plant immune system.

### RXLR9 suppresses callose deposition

**Figure 8:**
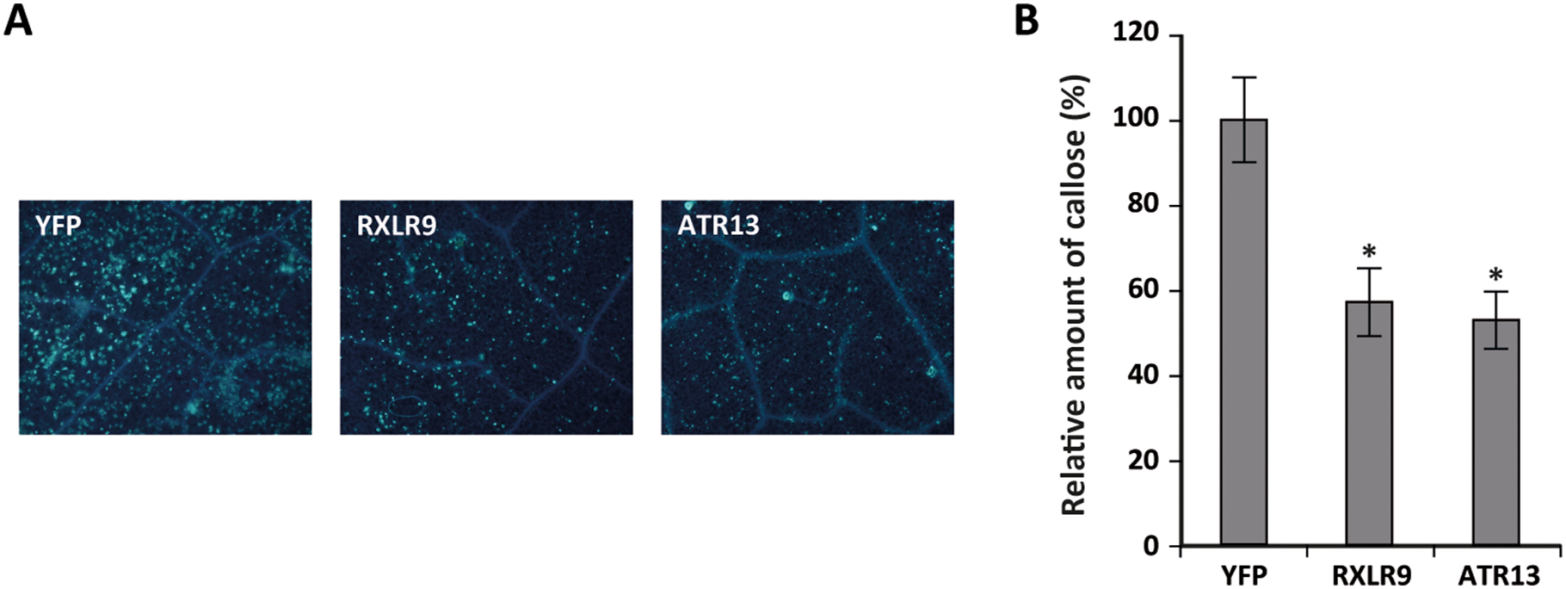
Effect of EDV-mediated delivery of Waco9 RXLR9 on *P. syringae* ΔCEL-induced callose deposition. Five-week-old Col-0 plants were infiltrated with *P. syringae* ΔCEL carrying YFP, RXLR9 or ATR13. After 12 h, leaves were harvested and stained with analine blue for the detection of callose deposition. Pictures were taken (A) and the number of callose spots was quantified (B). The combined results of three independent experiments are shown. Results represent mean ± SEM (*n* = 17–23) and asterisks indicate significant differences (ANOVA and Fisher’s LSD corrected for type I errors; *p*<0.05).

To further test the above observed RXLR trends independently of constitutive *in planta* expression, we used the bacterial effector detector vector (EDV) system. This system has been used successfully to deliver the *H. arabidopsidis* RXLR effector ATR13 into *A. thaliana* leaf cells [59]. It is based on the fusion of a candidate effector protein to the N-terminus of the type III secreted bacterial effector, AvrRPs4, allowing delivery of the effector into *A. thaliana* leaf cells by bacteria such as *P. syringae* pv. *tomato* DC3000 mutant ΔCEL, which strongly triggers MTI [54], [65]. Immune suppressive effects of the delivered putative effector can be tested by quantifying its effect on *P. syringae* ΔCEL-triggered callose deposition [39]. Waco9 RXLR29 has previously been shown to suppress pathogen-induced callose deposition [39]. Here, a different RXLR protein, RXLR9, was selected for analysis. RXLR9 was cloned in the EDV effector delivery system and expressed in the *P. syringae* ΔCEL mutant strain and delivered to *A. thaliana* by pressure infiltrating *P. syringae* ΔCEL (RXLR9) into leaves of Col-0 plants. As a negative control the same EDV system was used to deliver the YFP protein into plant cells, and EDV-ATR13 was used as a positive control [39], [59]. At 12 h after pathogen infiltration, the immune-suppressive effect of RXLR9 was evaluated by quantifying callose deposition in the infiltrated leaf tissue. Infiltration of Col-0 leaves with *P. syringae* ΔCEL (YFP) resulted in strong callose deposition at the site of tissue infiltration (Figure 8), confirming previous findings that *P. syringae* ΔCEL triggers a strong MTI response [39], [54], [65]. Also, infiltration of Col-0 with bacteria delivering ATR13 reduced the number of callose deposition sites, confirming the immune suppressive effect of ATR13 [59]. Infiltration with *P. syringae* ΔCEL delivering RXLR9 led to a reduction in callose production that was similar to that of ATR13. We therefore concluded that RXLR9 is able to suppress MTI.

## Discussion

When the genome sequences of different phytopathogenic oomycetes were unraveled, motif searches revealed that these pathogens possess large repertoires of putative effector genes. Different classes of effector candidate proteins have been identified, such as apoplastic effectors and host-translocated Crinklers and RXLR-effectors [23]. The last effector group is represented by proteins containing the RXLR motif in their amino acid sequence, which can be easily retrieved from the genome sequence [16]–[18], [66]. *H. arabidopsidis* strain Emoy2 has 134 predicted RXLR effectors, but for most of these information on expression and function is lacking. Cabral *et al*. [39] identified 18 RXLR proteins of the *H. arabidopsidis* strain Waco9 that are expressed during infection of *A. thaliana*. Here we describe the screening of these putative effectors for their impact on different components of the plant immune system.

### Conserved N-termini of RXLR proteins

Based on amino acid sequences, the set of 18 Waco9 RXLR proteins used in this study can be divided in several groups. Closer scrutiny of these groups revealed that the N-termini of the different RXLR proteins within one group are highly similar, while in most cases the C-termini show little similarity (Figure 1A). The set of 134 RXLRs identified in the *H. arabidopsidis* Emoy2 genome [18] display similar clustering, based on amino acid sequence similarity in the N-terminal protein domains (Figure 1B). Current evidence suggests that the C-terminus of RXLR proteins is important for their effector functions, while the N-terminus is involved in protein translocation [28], [29]. Many effector proteins are under selective pressure to evade detection by host immune receptors. During effector production and secretion in the *Plasmodium* parasite, the RXLR-related PEXEL motif is cleaved behind the leucine residue after which the C-terminal part of the protein is translocated into erythrocytes [67]. If the translocation system of *H. arabidopsidis* RXLR proteins works in a similar manner, the N-terminal part of the protein is not under diversifying selection driven by the host immune system. This might explain why RXLR proteins exhibit a relatively high level of amino acid conservation in the N-terminal part of the protein, while the C-terminal part following the RXLR motif shows relatively low level of sequence conservation.

### RXLR effectors modulate host defense responses

Transgenic plants expressing single *RXLR* genes did not show strongly enhanced susceptibility when infected with *H. arabidopsidis* (Figure 3). It is likely that the production of RXLRs by *H. arabidopsidis*, which include those that are ectopically expressed, is sufficient to render additional expression of particular effectors largely ineffective. The only Waco9 *RXLR* gene in our screen that has a homolog in *Phytophthora* species is *RXLR21* (Table 1). Since this *RXLR* gene is present in *Phytophthora* as well as *Hyaloperonospora* species, it is likely that *P. capsici* also possesses an *RXLR21* ortholog. Overexpression of *RXLR21* in *A. thaliana* did not influence the level of *P. capsici* disease. Whether this is due to production of a related RXLR protein by the pathogen itself remains unclear. However, overexpression of certain Waco9 RXLR proteins did affect the level of resistance against *P. capsici*. For example, overexpression of RXLR9, RXLR23 and RXLR29 reduced symptom development caused by *P. capsici* infection compared to wild-type Col-0 plants (Figure 4, Table 1). This is unexpected because RXLRs normally suppress host immune responses, resulting in enhanced symptom development. This paradox might be explained by the lifestyle of *P. capsici*, which consists of an initial biotrophic phase followed by a switch to necrotrophy. Disease was scored by determining the number of necrotic lesions on each plant at day 6 after inoculation and thus reflects the necrotrophic phase of the *P. capsici* infection. *H. arabidopsidis* is an obligate biotroph and suppressing host cell death is likely important for its survival. The RXLRs used in this study might therefore contribute to cell death suppression, which in the bioassays with the hemi-biotroph *P. capsici* would reduce necrotic lesion formation.

To test the influence of the Waco9 RXLRs on basal immune responses, RXLR transgenic lines were inoculated with a virulent bacterial strain of *P. syringae* pv. *tomato.* Overexpression of five of the 13 Waco9 RXLRs enhanced the susceptibility of *A. thaliana* to *P. syringae* infection in at least two independent experiments. Fabro *et al.* [36] tested 64 Emoy2 RXLRs and found that a similarly high proportion of the RXLR effectors altered the level of susceptibility of *A. thaliana* to *P. syringae* infection. Homologs of 4 Waco9 RXLRs were tested in this screening as well (RXLR6, RXLR16, RXLR21 and RXLR23). However, for none of these RXLRs a consistent effect on bacterial growth in Col-0 could be observed. Fabro *et al*. also found that, of the RXLRs that led to increased growth of *P. syringae,* 77% suppressed *P. syringae* ΔCEL-induced callose deposition. Callose deposition is a typical MTI response, suggesting that many RXLR effectors interfere with basal host immunity. In this study, flg22-induced growth reduction was used to monitor the effects of the different putative effectors on MTI. In some experiments, overexpression of Waco9 RXLRs resulted in an enhanced effect of flg22 treatment on growth reduction (RXLR3 and RXLR23 in the 50 nM treatment, RXLR4 and RXLR29 in the 500 nM treatment). Also, while the growth reduction generally did not differ between the Waco9 RXLR overexpressors and Col-0, a number of lines (RXLR3, RXLR4, RXLR16, RXLR17, RXLR21, RXLR23, RXLR29) displayed a trend towards an enhanced flg22 response (average growth reduction of fls22-treated plants larger than that in Col-0; Figure 6C and 6D). The enhanced flg22-mediated growth reduction is unexpected since repression of host basal immunity should attenuate flg22-mediated seedling growth inhibition. The cause of this puzzling finding remains unresolved, but one explanation might be that suppression of one defense signaling pathway by an RXLR effector leads to a compensatory upregulation of another pathway through cross-talk within the defense network [68]. Although not statistically significant, RXLR9 overexpressing plants consistently displayed a smaller reduction in fresh weight than Col-0 after treatment with 500 nM flg22 (Figure 6D). This is in line with our observations in the *P. syringae* bioassays that RXLR9 overexpressing plants have enhanced susceptibility to *P. syringae* infection (Figure 5) and that *P. syringae* ΔCEL delivering RXLR9 triggers less callose deposition (Figure 8). In contrast, plants expressing RXLR29, which has previously been shown to be able to suppress callose deposition [39], appear to have a stronger growth reduction after flg22 treatment. This might be explained by the fact that the different experiments, although both related to MTI, measure very different responses that are very likely regulated through different pathways.

### Multiple weak effectors might act strongly together

A number of RXLR proteins have previously been shown to severely alter host defense responses [36], [38]. In our study a number of RXLRs have clearly measurable effects on certain plant immune system outputs (e.g. RXLR9 and RXLR29 significantly affect susceptibility to *P. syringae* and *P. capsici*, respectively) but most RXLR effectors had no significant effect on the tested defense responses. Additionally, in some cases the variation between repeats was as important as the difference between the tested line and the control, making it difficult to draw conclusions based on the separate experiments. However, when data from all experiments were combined and analyzed in a multifactorial analysis, significant effects on host immunity were revealed for a relatively high number (12) of the 13 Waco9 RXLRs tested (Figure 7). Five functional effector groups were identified, but these did not correspond to the groups identified based on N-terminal protein sequence relationships. Also, Wang *et al*. [33] showed that ∼75% of 169 putative RXLR effectors of *P. sojae* influence host immunity, illustrated by their ability to suppress programmed cell death responses induced by the pro-apoptotic protein BAX. In addition, Fabro *et al*. [36] found that 72% of the tested *H. arabidopsidis* Emoy2 RXLRs suppress host immunity when delivered by the EDV system, resulting in enhanced growth of *P. syringae* in *A. thaliana*. In both studies, the RXLR effectors were identified in the pathogen genomes without prior information on expression patterns for these genes. In our study the Waco9 RXLRs tested were identified in an EST library of the *A. thaliana – H. arabidopsidis* Waco9 interaction [39] and are therefore likely to play a role in this plant-pathogen interaction. This might explain the relatively high level of RXLR proteins with some effect on host immunity in our screen. That no effects were observed for RXLR20 could mean that we should look at other defense responses of the plant to find an activity. However, it could also suggest that this protein targets other processes of its host, like nutrient transport.

In conclusion, the data presented in this paper suggest that a large number of the Waco9 *RXLR* genes that are expressed during infection of the host are likely to contribute to pathogen virulence. Further, the data obtained in the independent experiments show that many RXLR proteins have only weak effects on certain components of the plant immune system, which could only be revealed by combining the data of the different experiments. *H. arabidopsidis* contains 134 genes in its genome encoding potentially secreted proteins with an RXLR domain [18]. If several have minor activities on one or more components of the plant immune system, their concerted action might substantially modulate the host immune response. In this scenario, each RXLR protein with a minor activity might be disposed of or mutated without major loss of pathogenicity, offering a low risk evolutionary strategy.

## Supporting Information

Table S1 **Sequences of the primers used in this study.**
(DOCX)
