# Supplementary material for: Functional Analysis of Hyaloperonospora arabidopsidis RXLR Effectors
Source: PLoS One. 2014 Nov 6;9(11):e110624. doi: 10.1371/journal.pone.0110624 (PMC4222755; doi:10.1371/journal.pone.0110624)
Supplement: Table S1 — Sequences of the primers used in this study. (DOCX) [file pone.0110624.s001.docx]

| **Gene** | **Fw** | **Rv** |
| --- | --- | --- |
| *RXLR3* | CAC CAT GGG CTC AAC AAC TAT GGA C | TCA GAA ACG ATG CGG GCG |
| *RXLR4* | CAC CAT GGG CTC AAC AAC TAT GGG C | TCA GAA ACG ATG CGG GCG |
| *RXLR6* | CAC CAT GGA CCC ATC AAA CTC GGA | TTA CCC TAA TCC TGC GAG AAG |
| *RXLR9* | CAC CAT GCG CCT GTG TGC CAA C | CAC CAT GGA TGT TAC CCC AGT C |
| *RXLR13* | CAC CAT GGT CCC GAC TGC TG | ACC TAG CCA TCC TTG TGA C |
| *RXLR16* | CAC CAT GCT GCC AGC TCG CGC AG | TCA AAT CGC CGC ATT GAT GTC |
| *RXLR17* | CAC CAT GCT GCC AGC TCG CGC AG | TTA ATT CGA GTT CCT CTG GT |
| *RXLR19* | CAC CAT GGC CAC GGA GGC TGC TGG | CTA CGA GTG ACT AAC TGG AC |
| *RXLR20* | CAC CAT GGC CTC GGG ACT CGC GA | TTA CGC TTT TTT ACG CAT TAG AGC |
| *RXLR21* | TTA CCT ACC ATG ACC CGA C | CAC CAT GGT GTC CAC GGC CAC GAA C |
| *RXLR22* | CAC CAT GGA CCC ATC AAA CTC GGA C | CTA CTC GAG CGG AGC AAG |
| *RXLR23* | CAC CAT GGT CCC GAC TGC TGG TAA TC | CGT TCA TCT CTG GAT TGT ATC |
| *RXLR29* | CAC CAT GGA GGT GGT CCT GAT C | TTA CTT GCC AGG ACG CGC |
| *ACTIN* | AAT CAC AGC ACT TGC ACC A | GAG GGA AGC AAG AAT GGA AC |

**Supplementary Table S1**: Sequences of the primers used in this study.
